# RAPIDR: an analysis package for non-invasive prenatal testing of aneuploidy

**DOI:** 10.1093/bioinformatics/btu419

**Published:** 2014-07-01

**Authors:** Kitty K. Lo, Christopher Boustred, Lyn S. Chitty, Vincent Plagnol

**Affiliations:** ^1^University College London Genetics Institute, University College London, UK and ^2^North East Thames Regional Genetics Service, Great Ormond Street Hospital NHS Foundation Trust, London, UK

## Abstract

Non-invasive prenatal testing (NIPT) of fetal aneuploidy using cell-free fetal DNA is becoming part of routine clinical practice. RAPIDR (Reliable Accurate Prenatal non-Invasive Diagnosis R package) is an easy-to-use open-source *R* package that implements several published NIPT analysis methods. The input to RAPIDR is a set of sequence alignment files in the BAM format, and the outputs are calls for aneuploidy, including trisomies 13, 18, 21 and monosomy X as well as fetal sex. RAPIDR has been extensively tested with a large sample set as part of the RAPID project in the UK. The package contains quality control steps to make it robust for use in the clinical setting.

**Availability and implementation:** RAPIDR is implemented in *R* and can be freely downloaded via CRAN from here: http://cran.r-project.org/web/packages/RAPIDR/index.html.

**Contact:** kitty.lo@ucl.ac.uk

**Supplementary information:** Supplementary data are available at *Bioinformatics* online.

## 1 INTRODUCTION

Since the first demonstration of the use of cell-free DNA (cfDNA) to detect aneuploidy (Chiu *et al.*, 2008; Fan *et al.*, 2008), many groups around the world have reported large studies showing high sensitivities and specificities for the detection of trisomies 13, 18 and 21 (Jiang *et al.*, 2012; Palomaki *et al.*, 2011; Sehnert *et al.*, 2011). In contrast to invasive methods such as amniocentesis, non-invasive prenatal testing (NIPT) uses cfDNA in maternal plasma and does not present a risk of miscarriage. Currently, outside the context of research studies, NIPT for aneuploidy is only available from commercial providers.

To perform NIPT, cfDNA is extracted from maternal plasma and sequenced using short reads and high-throughput DNA sequencing. Sequenced reads are aligned to the reference human genome using a short read aligner. Aneuploidy is called if the number of reads mapped to each chromosome differs from its expectation based on a comparison with a set of baseline samples. The general approach is usually referred to as a read depth strategy.

A number of factors influence test performance, for example the fetal fraction (i.e. the proportion of reads originating from the fetus), and other technical parameters such as read length and coverage (Fan and Quake, 2010). The choice of sequencing parameters needs to be balanced against costs. Previous studies have shown that an acceptable level of test sensitivity can be reached with relatively short read lengths (36–52 bp) and modest coverage (2–10 million reads per sample) (Liang *et al.*, 2013; Sehnert *et al.*, 2011).

Here, we present a flexible and robust *R* package for NIPT analysis called RAPIDR (Reliable Accurate Prenatal non-Invasive Diagnosis R package). RAPIDR combines several analytical techniques that have been proposed for NIPT analysis and has been tested with a large sample set from the RAPID project (NIHR funded project to evaluate the use of NIPT). RAPID validation results can be found in the Supplementary Material (Supplementary Figs S1–S4, Supplementary Text S1 and Supplementary Tables S1 and S2).

## 2 CORRECTION FOR TECHNICAL ARTEFACTS

**Fig. 1.**
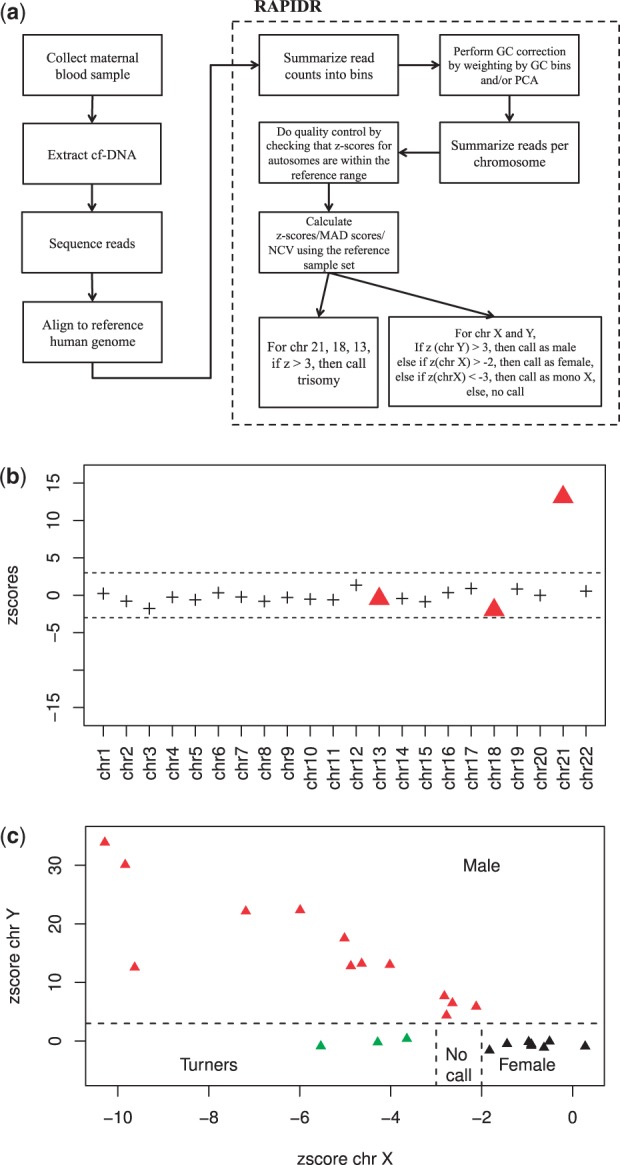
(**a**) Workflow of NIPT; (**b**) The *z*-scores across all the autosomes for a T21 sample, the triangles highlight chromosomes 13, 18 and 21 for which we test for aneuploidy; (**c**) Visual display of the criteria used to call fetal sex and chromosome aneuploidy

The NIPT workflow is illustrated in Figure 1a. RAPIDR handles the processing steps after reads have been sequenced and aligned, i.e. it starts from the BAM files. It uses the Rsamtools and the GenomicRanges R packages to parse the BAM files and count the number of reads in predefined bins (20 kb as default bin length, which must be sufficiently large to obtain at least 20 reads per bin).

Raw read counts from high-throughput sequencing technologies typically do not follow theoretical counting statistics. The excess variance, introduced in part by the polymerase chain reaction steps, is correlated with the GC content of the DNA sequence. Various methods have been proposed to correct for these biases. RAPIDR implements two methods from the literature: the normalized chromosome value (NCV) method (Sehnert *et al.*, 2011) and weighting of counts by GC content bin (Fan and Quake, 2010). These methods require a reference sample set, in contrast to the open source tool described by Straver *et al.* (2014). NCV computes the ratio between the counts mapped to the chromosome of interest and another chromosome. The chromosome used in the denominator is chosen to minimize the variance of the NCV ratios. In the GC bin weighting method (Fan and Quake, 2010), we first determine the read count in each bin, and then compute the average count as a function of GC content (using discrete intervals of 0.5% for GC proportion). Counts in each bin are then weighted by $W_{i}=\bar{M}/M_{i}$, where $\bar{M}$ is the average counts across all bins and *M_i_* is the average count in bin *i*. We also implemented a third method for GC correction based on principal component analysis (PCA), which as far we know, has not been used in the context of NIPT. In the PCA method, we first use the euploid samples in the baseline set to find the principal components by treating the count ratios in each bin as a variable. Then, we regress the count ratios in each bin with the first 10 principal components, with the assumption that they represent systematic noise. We find that PCA provides higher sensitivity than GC bin weighting correction in exchange for lower specificity (Supplementary Table S1).

Low complexity and repetitive regions are common in the human genome, which complicates the alignment of short sequencing reads. We recommend only keeping uniquely mapped reads. RAPIDR provides the option to mask out parts of genome known to be of low complexity. By default, it excludes bins with abnormally high read counts from the analysis.

## 3 CALLING OF ANEUPLOIDY AND FETAL SEX

Once artifacts have been removed from the read counts, the next step is for RAPIDR to create a baseline using a set of reference samples with known outcomes and fetal sex. For each sample, RAPIDR calculates the ratio of reads mapped to each chromosome compared with the reads mapped to all the autosomes (call this ratio *r_ij_* for chromosome *i* and sample *j*). Using the set of reference samples without known aneuploidy, it then computes the mean ($\mu_{i}$) and SD ($\sigma_{i}$) of *r_ij_* for chromosomes 21, 18 and 13. To call chromosomal abnormalities, it calculates a z-score *z_ij_* for each sample–chromosome pair (Fig. 1b). A trisomy is called if that *z*-score exceeds a specified threshold (typically *z* > 3). We estimate that a relatively small reference sample set is sufficient to provide accurate results, however a bigger reference sample set would ensure any inter-run variabilities are captured. (Supplementary Text S2).

An important issue is technical sample-to-sample variability. If a sample is technically too distinct, it will not be appropriate to include it in the baseline. We propose quality control checks to ensure that such technical issues are not affecting the calls. One useful check is to make sure that the z-score for all autosomes (aside from the chromosomes for which we suspect aneuploidy) fall between the expected range. RAPIDR provides functions to report and visualize this easily, and Figure 1b shows the quality control output from one sample.

A different and slightly more complex process is required to determine fetal sex and to call monosomy X abnormalities. Given an unknown sample, the *z*-score is calculated for chr X and chr Y (*z_X_* and *z_Y_*), with the following rules being applied sequentially—if *z_Y_* > 3, the sample is called male; if $z_{X}>-2$, the sample is called female; if $z_{X}<-3$, the sample is called as monosomy X; finally, if *z_X_* is between −2 and −3, then the sample sex is called as unknown (Fig. 1c). To improve the accuracy of fetal sex calling, we also excluded bins in chr Y with a high number of counts mapped from females, e.g. the pseudo-autosomal region of chr Y.

For euploid males, RAPIDR also estimates the fetal fraction using the method from Rava *et al.* (2014). Fetal fraction is an important determinant of NIPT sensitivity, but it cannot be measured directly using the protocol we described.

## 4 CONCLUSION

In this article, we described the RAPIDR package for NIPT of fetal aneuploidies. RAPIDR has been validated using >700 samples and delivers an acceptable level of specificity and sensitivity for T21, T18, T13 and monosomy X. The system requirement for RAPIDR is modest (Supplementary Text S3). Future work on RAPIDR will include evaluating strategies to detect sub-chromosomal abnormalities.

*Funding*: This manuscript presents independent research funded by the National Institute for Health Research (NIHR) under the Program Grants for Applied Research Program (RP-PG-0707-10107) (the RAPID project) and the NIHR Biomedical Research Centre at Great Ormond Street Hospital. L.S.C. is partially funded by the Great Ormond Street Hospital Children’s Charity. The views expressed are those of the authors and not necessarily those of the NHS, the NIHR or the Department of Health.

*Conflict of interest*: none declared.

## Supplementary Material

Supplementary Data
