# Supplementary material for: RAPIDR: an analysis package for non-invasive prenatal testing of aneuploidy
Source: Bioinformatics. 2014 Jul 1;30(20):2965–7. doi: 10.1093/bioinformatics/btu419 (PMC4184262; doi:10.1093/bioinformatics/btu419)
Supplement: Supplementary Data [file supp_30_20_2965__index.html]

RAPIDR: an analysis package for non-invasive prenatal testing of aneuploidy — RAPIDR: an analysis package for non-invasive prenatal testing of aneuploidy — RAPIDR: an analysis package for non-invasive prenatal testing of aneuploidy — RAPIDR: an analysis package for non-invasive prenatal testing of aneuploidy — Supplementary Data 

# RAPIDR: an analysis package for non-invasive prenatal testing of aneuploidy

## Supplementary Data

files

**Files in this Data Supplement:**

- Supplementary Data - docx file
