# Supplementary material for: RAPIDR: an analysis package for non-invasive prenatal testing of aneuploidy
Source: Bioinformatics. 2014 Jul 1;30(20):2965–7. doi: 10.1093/bioinformatics/btu419 (PMC4184262; doi:10.1093/bioinformatics/btu419)
Supplement: Supplementary Data [file supp_btu419_supp_materials.docx]

**Supplementary material**

**Text S1. Validation results**

The validation data set consists of 732 samples collected as part of the RAPID project in the UK ([www.rapid.nhs.uk](http://www.rapid.nhs.uk)). Cell-free DNA are extracted from maternal plasma using standard protocol and sequenced with the Illumina HiSeq2500 machine. Reads are single-ended and are either 36 bp or 50 bp in length. Total number of mapped reads range from 2 million to 13 million, which implies that the coverage ranges between 0.02 and 0.22X. Two samples with fewer than 2 million mapped reads were excluded from further analysis.

Illustrative results from our validation dataset, comparing sensitivity and specificity of the different normalization methods, using the Z-score are shown in Tables S1 & S2. RAPIDR provides a number of further analysis options, e.g. using Median Absolute Deviation-score and masking counts from repetitive regions that are not presented here (manuscript in preparation). The different analysis options made available in RAPIDR enable users to determine the most appropriate method for their specific samples (e.g. which may have differing GC biases depending on the sequencing chemistry used) and for their specific outcomes of interest.

**Table S1.**

Comparison of sensitivity and specificity for calling T21, T18, T13 and monosomy X using three different count normalization methods provided by RAPIDR.

| **Aneuploidy** | **Normalization method** | **True positives** | **True negatives** | **False positives** | **False negatives** | **Sensitivity** | **Specificity** |
| --- | --- | --- | --- | --- | --- | --- | --- |
| T21 | GC correction | 100 | 626 | 2 | 2 | 98.0% | 99.7% |
|  | NCV | 101 | 627 | 1 | 1 | 99.0% | 99.8% |
|  | PCA | 100 | 627 | 1 | 2 | 98.0% | 99.8% |
| T18 | GC correction | 37 | 691 | 0 | 2 | 94.9% | 100% |
|  | NCV | 38 | 690 | 1 | 1 | 97.4% | 99.9% |
|  | PCA | 38 | 691 | 0 | 1 | 97.4% | 100% |
| T13 | GC correction | 14 | 716 | 0 | 0 | 100% | 100% |
|  | NCV | 14 | 714 | 2 | 0 | 100% | 99.7% |
|  | PCA | 14 | 711 | 5 | 0 | 100% | 99.3% |
| Monosomy X | GC correction | 16 | 704 | 2 | 8 | 66.7% | 99.7% |
|  | NCV | 15 | 704 | 2 | 9 | 62.5% | 99.7% |
|  | PCA | 19 | 705 | 1 | 5 | 79.2% | 99.9% |

**Table S2.**

Comparison of sensitivity and specificity for calling fetal gender using three different count normalization methods provided by RAPIDR. Of the 730 samples in our dataset, we excluded 48 samples did not have a reported gender, and 24 monosomy X samples, which leaves 658 samples reported in the table below. Of the 658 samples, 313 are females and 345 are males.

| **Normalization method** | **Fetal Sex** | **Called as Male** | **Called as Female** | **Called as Monosomy X** | **No call** | **Accuracy** |
| --- | --- | --- | --- | --- | --- | --- |
| GC correction | Male | 333 | 8 | 0 | 4 | 96.5% |
|  | Female | 1 | 305 | 2 | 5 | 97.4% |
| NCV | Male | 336 | 6 | 1 | 2 | 97.4% |
|  | Female | 0 | 308 | 1 | 4 | 98.4% |
| PCA | Male | 341 | 2 | 0 | 2 | 98.8% |
|  | Female | 0 | 305 | 1 | 7 | 97.4% |

**Figure S1.** Chr13 z-scores output from RAPIDR based on 732 samples with known outcomes. The count normalization method used was GC bin weighting. Red triangles are trisomy 13 cases, blue triangles are trisomy 21 case, green triangles are trisomy 18 cases, cyan triangles are monosomy X cases and black triangles are euploids. The dotted line shows the z = 3 cutoff used in RAPIDR to call trisomies.

**Figure S2.** Same as Figure 1 but for chromosome 18.

**Figure S3.** Same as Figure 1, but for chromosome 21

**Figure S4.** Plot of z-scores of chromosome X versus z-scores for chromosome Y for 732 samples with known outcomes. GC bin weighting normalization was used. Red triangles are males, black triangles are females, and green triangles are monosomy X cases.

**Text S2. Size of reference set**

An estimate for the appropriate size of the reference set can be obtained through a traditional power study. Taking T21 as an example, we are interested in the proportion of reads mapping to chromosome 21 ($r_{21})$, computed after applying GC correction to each bin’s read count. Based on our set of reference samples, the mean of $r_{21}$ is 0.0128 and the standard deviation of $r_{21}$, denoted by $\sigma_{21}$, is $5.9190\times{10}^{-5}$. Note that $\sigma_{21}$depends on the read depth and the bias correction technique used hence our sample size calculation here is illustrative for our validation set.

For a T21 sample, $r_{21}$ would be multiplied by a factor of 1 + FF/2. As an illustration of a low fetal fraction case of 3%, the multiplicative factor would be 1.015. For the test sample, the standard deviation is set, but for the reference set this number is inversely proportional to the square root of the size of the reference set. Under the null hypothesis that the test sample has no aneuploidy, the difference between the reference $r_{21}$ and the test $r_{21}$ would be zero, but for the alternative hypothesis, i.e. a trisomy sample, the difference would be $r_{21}\times FF/2$. The variance of this difference ${\sigma_{diff}}^{2}$ is the sum of the variance of the test ratio and the reference ratio, i.e. ${\sigma_{21}}^{2}+\frac{{\sigma_{21}}^{2}}{N}.$ We can estimate the detection power at different levels of fetal fraction by calculating the probability that the ratio difference under the alternative hypothesis is greater than ${3\sigma}_{diff}$ (using a z-score threshold of 3). Figure S5 shows the detection power versus the reference sample size at three different levels of fetal fraction. It can be seen that detection power rises rapidly with reference sample size and the fetal fraction is a strong determinant of detection power.

**Figure S5.** Detection power versus size of reference set at fetal fraction of 10%, 5% and 3%.

**Text S3. Performance criteria**

The system requirement of RAPIDR depends on the choice of bin size, the number of samples in the reference set, and the analysis method used. We tested RAPIDR with our validation set of 732 samples, bin size of 20kb, on an AMD64 2.8GHz machine and found that the performance and memory requirement are as follows:

| **Normalization method** | **Time (minutes)** | **Memory (Gb)** |
| --- | --- | --- |
| NCV | 2.5 | 5 |
| GC bin weighting | 13 | 6 |
| PCA | 4.5 | 7 |
